# Necrotizing fasciitis following measles vaccine administration: a case report

**DOI:** 10.1186/s12879-019-4158-1

**Published:** 2019-06-14

**Authors:** E. E. Isere, A. A. Fatiregun, O. A. Olubosede, M. O. Dosumu, E. O. Bello

**Affiliations:** 1 World Health Organization, Ondo State Field office, Akure, Nigeria; 2 Department of Paediatrics, State Specialist Hospital, Akure, Ondo State Nigeria

**Keywords:** Adverse event following immunization, Necrotizing fasciitis, Measles vaccination

## Abstract

**Background:**

The occurrence of adverse events following immunization (AEFI) in national immunization programmes is very rare; however, if they occur causality assessment is conducted to identify the associated cause. In the report, we describe a case of severe necrotizing fasciitis in the left arm of a 9-month old boy following administration of the measles vaccine.

**Case presentation:**

A 9-month old boy presented with swelling on the left upper arm and adjoining the chest area, low-grade continuous fever, frequent passage of loose watery stool and persistent cries 24 h after measles vaccine was administered on the left upper arm. On examination, he was mildly pale, febrile, anicteric. Extensive erythema of the left upper arm occurred thereafter with extensive scalded skin lesions involving the deltoid area, the upper chest wall and arm. This was followed by desquamation of the affected areas and severe necrosis. A diagnosis of severe necrotizing fasciitis was made. A causality assessment was conducted by the state AEFI committee using the detailed AEFI investigation forms to identify the cause of the incidence.

**Conclusion:**

Here we present a rare case of necrotizing fasciitis which could have been caused by incorrect use of reconstituted measles vaccine. Hence we recommend training of routine immunization service providers on proper vaccine management as well as intensified supervision of immunization sessions.

## Background

Necrotizing fasciitis (NF) is a the life threatening soft-tissue infection, that is characterized by inflammation and subsequent necrosis of the muscles, fascia, subcutaneous fat and the epidermis in some cases [1–3]. Initial symptoms are nonspecific: the most frequently reported signs are fever, tenderness, erythema, and pain [3, 4]. Commonly recognized predisposing events include surgery, trauma, ruptured varicella blisters, and intramuscular injection. The common predisposing factors in newborns include omphalitis, circumcision, bullous impetigo, rectal temperature measurement and electrode placement for vital signs monitoring [3–7].However, few cases of post-vaccination NF, an adverse events following immunization (AEFI), have been reported in infants in literature [3, 4].

Measles is an acute viral infectious disease and an important cause of childhood morbidity and mortality [8]. Routine vaccination of children at 9 months according to the national immunization schedule of Nigeria with the live-attenuated measles vaccines has substantially reduced the risk of measles infection and measles deaths annually [8–10]. However, in some very rare occasion, cases of adverse events following immunization may occur. AEFI are any untoward medical occurrences which follow immunization and which do not necessarily have a causal relationship with the use of the vaccine [11]. These adverse events may be any unfavourable sign, abnormal laboratory finding, symptom or disease occurring after immunization [11].

In this report, we present a case of necrotizing fasciitis in a 9-month old child after receiving the measles vaccine, and the outcome of causality assessment.

## Case presentation

A 9-month old boy presented at a hospital in a south western state of Nigeria, with a swollen left upper arm adjoining the chest, low-grade continuous fever (38.1 °C), frequent passage of loose watery stool and persistent cries for more than 3 h. Child had been immunized about 24 h earlier. The mother reported that the symptoms were observed 2 h after the child was vaccinated with the measles vaccine at a private hospital. The child was one of three children reported to have been vaccinated with measles vaccine at a private hospital during the immunization clinic session.

On examination, he was mildly pale, febrile and anicteric. He was moderately dehydrated; mildly dyspnoeic with normal heart sound, heart rate of 148 beats/ min, breath sound was vesicular and respiratory rate of 54 cycles per minute. He was well nourished as the weight was appropriate for age. There was extensive swelling with skin discolouration (hyperemia) involving the entire left upper arm, sparing the distal third of the forearm and hand. There was also swelling of the upper part of the anterior chest wall. The swelling was firm and mildly tender. There was no history of adverse reaction to immunization or any form allergic reaction.

**Fig. 1:**
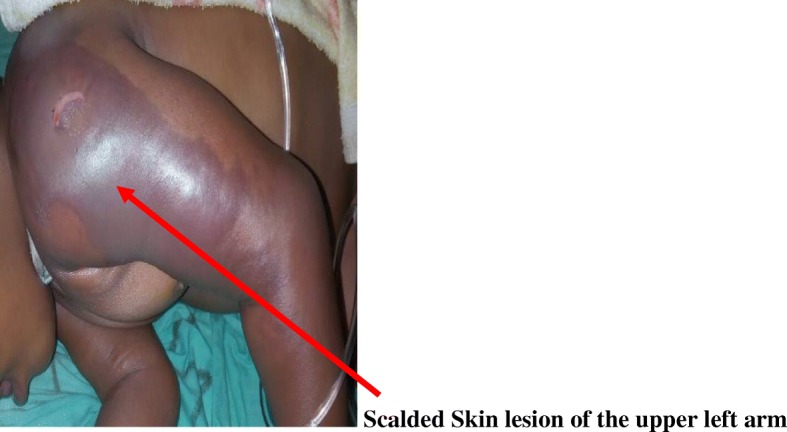
Scalded Skin lesion of the upper left arm

**Fig. 2:**
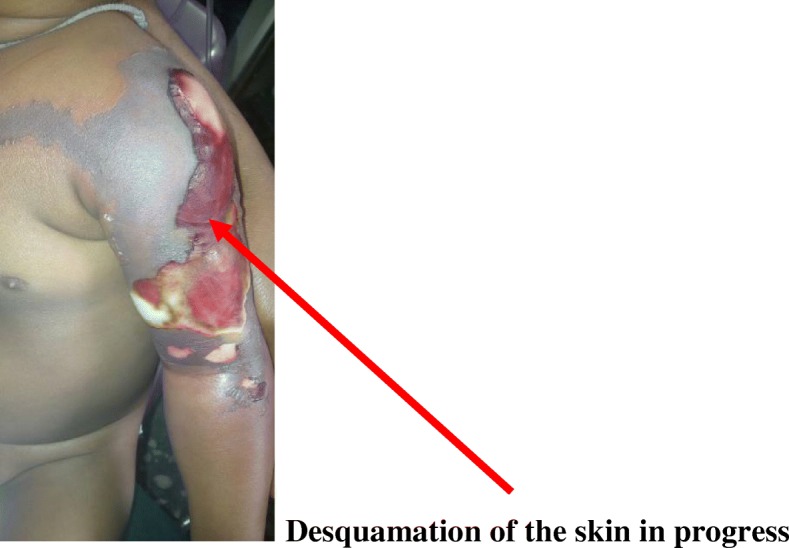
Desquamation of the skin in progress

**Fig. 3:**
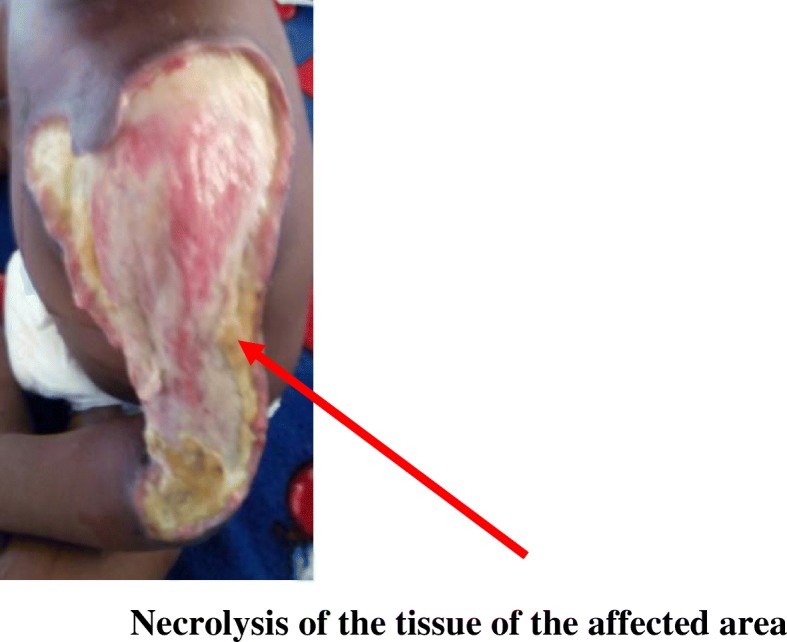
Necrolysis of the tissue of the affected area

A day after admitting the child, extensive erythema of the left upper arm and anterior area of the chest was observed with extensive scalded skin lesion involving the deltoid area, the upper chest wall and arm (Fig. 1). Desquamation of the affected areas was observed presenting like severely burned skin from a hot liquid. There was darkening and hardening of the skin over the affected area on the arm with eventual severe necrosis up to a depth of about 5 mm thereafter (Figs. 2 and 3). A diagnosis of severe necrotizing fasciitis was made.

Radical debridement of necrotic tissues was carried out under general anaesthesia. Child was also transfused with blood. Daily dressing of the wound was done and antibiotics administered were intravenous metronidazole (20 mg/ kg/ day in 3 divided doses) and ceftazidime (100 mg/ kg/ day in 3 divided doses). Child was referred to University College Hospital, Ibadan, a teaching hospital in a neighbouring state where skin grafting was performed. Presently, child have recovered and he is fully healthy.

### Causality assessment

A causality assessment was conducted by the state AEFI committee using the detailed AEFI investigation forms using WHO AEFI causality assessment methodology [12, 13].Visits were made to the private hospital where the child was reported to have received the vaccine. The routine immunization focal person in the facility was interviewed. Assessment of available cold chain devices for vaccine storage was also carried out. The knowledge and skills of health workers in vaccine handling, management and administration were assessed [14–16]. In addition, the caregivers of two other children immunized during the session were recalled and interviewed. The case of interest was the first child to be vaccinated with measles vaccine during the immunization clinic while the second child, a 9 months old female who received vaccination from the same measles vial had fever and abscess formation at the site of immunization only however, the third child who was also vaccinated during the immunization clinic was healthy and without symptoms. The third child was found to be vaccinated with measles vaccine from a newly reconstituted measles vaccine vial different from the measles vaccine vial used for the other two children on the day of the immunization clinic. Incision and drainage procedure was carried out for the second child with wound dressing conducted for two weeks who thereafter recovered fully.

The findings from the investigation indicated that a programmatic error may have been responsible for the reactions.We found that two children were vaccinated with a measles vaccine that have been reconstituted for a period of > 6 h. The measles vaccine administered to these children was reconstituted 7 days ago and used during the previous immunization clinic with the left-over stored in a refrigerator within the hospital. This was due to poor knowledge and skill in vaccine management and administration among health workers who administered the vaccine. Other key issues identified includes poor documentation of vaccination activities using the recommended data management tools resulting in difficulty to tracked other children vaccinated with other vaccines for further investigation and poor vaccine storage system at the private hospital as the hospital lacks the recommended Solar Direct Drive (SDD) refrigerator for proper vaccine storage. Also, effort to retrieve the samples of the left-over doses of the vaccine in the opened vials for laboratory investigation proved abortive as the used/empty vial of the vaccine was said to have been discarded by the health workers immediately after the immunization clinic. Furthermore, blood samples collected from the child with NF by the attending physician during the preliminary case management at a local hospital for microbiological culture investigation shows contamination of culture plate as samples were not properly stored during the culture process due to lack of the required facility to perform the test at the hospital.

## Discussion and conclusion

Necrotizing fasciitis in infants has been well documented in literature with several predisposing factors identified [5–7]. However, in this infant, no obvious predisposing factors other than the measles vaccination in the affected arm could be identified.

Several clinical manifestations in this case are consistent with what have been reported by previous authors. Thema et.al, [3] in a case report of NF following BCG vaccination reported fever, pain and erythematous swelling of the outer aspect of the middle third of the left arm with sloughing and necrosis. Similarly, Lemerechal et.al [4] reported fever, mildly painful swelling and erythema on the affected area with local necrosis of ~ 3 mm in diameter. The clinical sign of passage of loose stool and moderate dehydration in this case is not consistent with previous reports. However, Zundel et.al [17] have reported that clinical signs of NF in infants most times are non-specific with fever, erythema, pain, tenderness as the most frequently reported symptoms.

Broad spectrum antibiotic is recommended for use in NF because of the wide range of organisms such as streptococci, staphylococci, gram negative rods and anaerobes that could be involved. [18, 19]. Although many studies have favoured the use of antibiotic combinations that included clindamycin [2–4], some other studies did not while one study found that it was not sensitive and had to be changed with the availability of sensitivity report [4]. The antibiotic used in the case was intravenous metronidazole and ceftazidime; and child responded with good clinical improvement. Clindamycin was not used because it was not readily available in our locality.

From our findings from the causality assessment, we presumed that contamination of the vial of vaccine with *Staphylococcus aureus* and possibly other organism particularly group A *Streptococcus* (GAS) should have occurred*.* According to World Health Organization (WHO), all reconstituted vaccines are expected to be discarded after 6 h or at the end of every immunization session or whichever comes first [20]. It is well documented in literature that when reconstituted vaccines are stored beyond 6 h of reconstitution contrary to the recommendation, contamination with *Staphylococcus aureus* and other organisms such as the GAS can occur [20, 21]. This is because these vaccines do not contain preservatives [15]. It has been reported that when a reconstituted vaccine is contaminated, the organism grows rapidly and produces toxins that could be responsible for the type of necotizing lesion observed in the present case [1, 20, 21].

Incorrect use of reconstituted measles vaccine could be responsible for the case of necrotizing fasciitis in this report. Hence, we recommend regular training of immunization providers on vaccine handling, management and administration. We also recommend intensified supportive supervision, especially to private hospitals during the conduct of routine immunization sessions to avert future immunization programme error.

## Data Availability

Data sharing is not applicable to this article as no datasets were generated or analysed during the current study.

## Abbreviations

AEFI: Adverse Events Following Immunization
BCG: Bacille de Calmette et Guérin
GAS: Group A *Streptococcus*
NF: Necrotizing fasciitis
WHO: World Health Organization
